# Progress in the functional modification of graphene/graphene oxide: a review

**DOI:** 10.1039/d0ra01068e

**Published:** 2020-04-17

**Authors:** Wang Yu, Li Sisi, Yang Haiyan, Luo Jie

**Affiliations:** School of Mechanical Engineering, Xihua University 9999 Hongguang Avenue, Pidu District Chengdu City Sichuan Province 611730 P. R. China; School of Automation Engineering, University of Electronic Science and Technology of China China; School of Materials Science and Engineering, Southwest Petroleum University China; Patent Examination Cooperation Sichuan Center of the Patent Office China; Petrochina Southwest Pipeline Company China

## Abstract

Graphene and graphene oxide have attracted tremendous interest over the past decade due to their unique and excellent electronic, optical, mechanical, and chemical properties. This review focuses on the functional modification of graphene and graphene oxide. First, the basic structure, preparation methods and properties of graphene and graphene oxide are briefly described. Subsequently, the methods for the reduction of graphene oxide are introduced. Next, the functionalization of graphene and graphene oxide is mainly divided into covalent binding modification, non-covalent binding modification and elemental doping. Then, the properties and application prospects of the modified products are summarized. Finally, the current challenges and future research directions are presented in terms of surface functional modification for graphene and graphene oxide.

## Abstract 2

Graphene and graphene oxide have attracted tremendous interest over the past decade due to their unique and excellent electronic, optical, mechanical, and chemical properties.

## Graphical abstract

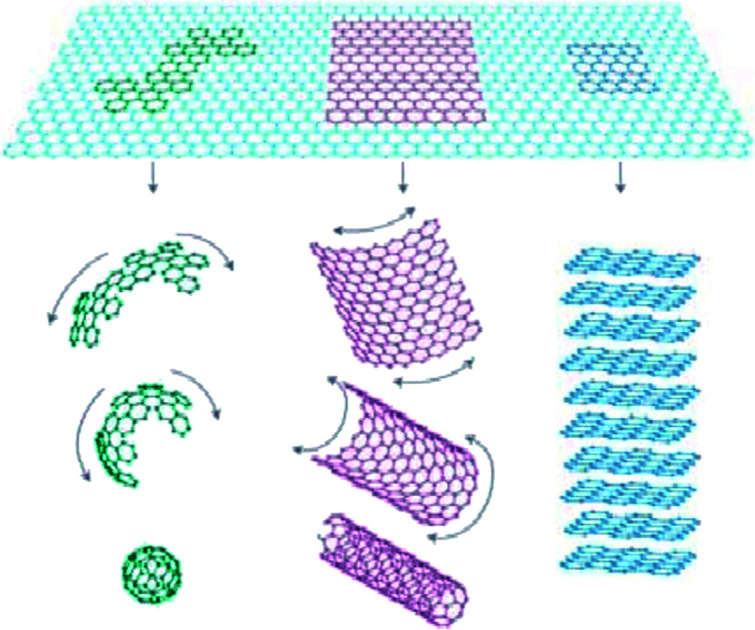

## 1. Introduction

**Fig. 1:**
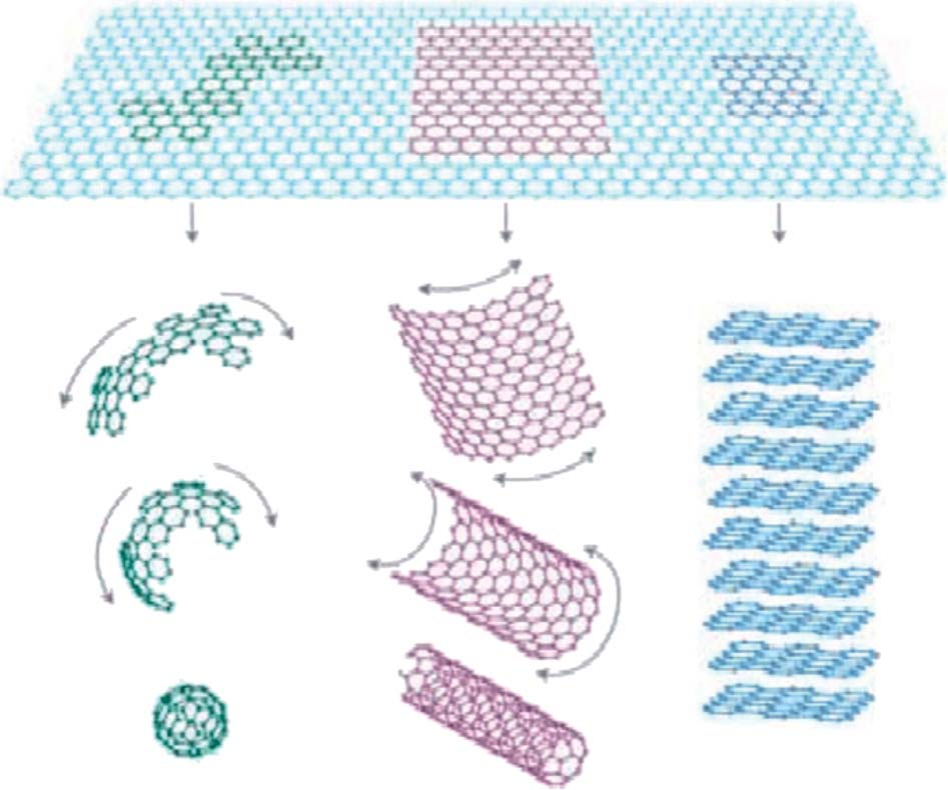
Carbon allotropes: graphene to fullerene, nanotubes and graphite. This figure has been reproduced from ref. 19 with permission from Springer Nature, Copyright 2007.

Graphene is a carbon material,^1^ and in 1985, Robert Curl *et al.*^2^ prepared a C60 product. However, four years later, Krätschmer^3^ confirmed the cage structure of C60-fullerene. In 1991, Nippon Electric Company (NEC) Ltd. first reported carbon nanotubes and expanded the carbon material family.^4^ In 2004, Novoselov *et al.*^5^ successfully separated graphene from the monolithic state using a micro-computer peeling method, which challenged the scientific understanding of two-dimensional crystals. The structure of graphene is shown in Fig. 1, which is composed of a layer of independent sp^2^ hybrid carbon atoms. It is a two-dimensional carbonaceous material with a hexagonal honeycomb crystal structure. To now, graphene is the thinnest and strongest nanomaterial, with a sheet thickness of 0.34 nm.^6^ Each carbon atom in graphene is bonded to three adjacent carbon atoms through a σ bond. The remaining p electrons most likely form a π bond with the surrounding atoms due to their failure to form a bond, and the bonding direction is perpendicular to the graphene plane. The structure of graphene is very stable, and its C–C bond length is only 0.142 nm.^7^ The connection between each carbon atom of graphene is very strong. When an external force is applied to graphene, the atomic surface inside it is deformed and further bent to offset the external force. Thus, there is no rearrangement and misalignment between the carbon atoms, maintaining a consistently stable structure.^8^ When the electrons of graphene move in the internal orbit, there is no scattering phenomenon due to the interference of foreign atoms or lattice defects.^9,10^ This unique lattice structure gives graphene various excellent properties. Nowadays, there are numerous methods for the preparation of graphene, but the main preparation methods include mechanical stripping, liquid phase stripping, chemical vapor deposition, epitaxial growth and redox methods.^11^ Recently, significant research has been conducted on graphene quantum dots,^12–14^ and carbon doped with other elements, molecules or organic materials.^15–18^

Compared with graphene (G), graphene oxide (GO) has the advantages of low production cost, large-scale production, and easy processing. It is often used as a precursor for the preparation of reduced graphene oxide (RGO).^20^ In recent years, with the further study of GO, scientists have found that it also has excellent properties with rich active oxygen-containing functional groups.^21^ These oxygen-containing groups or reduced doping elements can be used as catalytic active centers for covalent/non-covalent modification design according to the requirements of specific application fields. In addition, the presence of oxygen-containing groups also broadens the interlayer gap of graphene oxide. It can be functionalized by small molecules or polymer intercalations. At present, great progress has been achieved in the functionalization of graphene oxide. It has been applied in the fields of desalination,^22^ drug delivery,^23^ oil–water separation,^24^ immobilization catalysis,^25^ solar cells,^26^ energy storage,^27^ healthcare,^28,29^*etc.*

However, the single component graphene material has certain limitations, such as weak electrochemical activity, easy agglomeration and difficult processing, which greatly limit the application of graphene. Therefore, functional modification of graphene and graphene oxide is crucial to expanding their application. The functionalization of graphene and graphene oxide is based on the further modification of their intrinsic structure. Herein, we introduce the functional modification methods based on the intrinsic structural chemical bonds and functional groups of graphene and graphene oxide. Firstly, we introduce the basic structure and properties of graphene and graphene oxide, and then their functionalization based on their surface structure features, which is divided into three types, functional modification of covalent bond bonding, functional modification of non-covalent bond action and element doping modification. Subsequently, we categorize and systematically summarize the reaction process and reaction conditions of typical reaction types and their research methods. Finally, the future of surface functionalization of graphene and graphene oxide is presented.

## 2. Structure and properties of graphene/graphene oxide

**Fig. 2:**
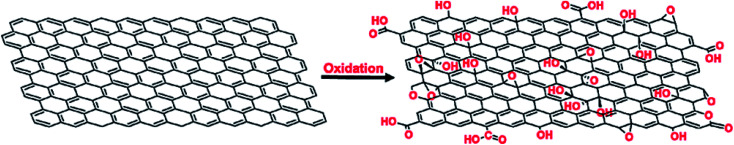
Oxidation of graphene sheet to form graphene oxide. This figure has been reproduced from ref. 43 with permission from *Chem. Rev.*, Copyright 2016.

Graphene has a planar hexagonal lattice structure. There are 4 valence electrons per carbon atom, including 3 electrons (2s electron, 2p_*x*_ electron and 2p_*y*_ electron), forming plane sp^2^ hybrid orbitals. The remaining orbital electron forms a large π bond, and this electron can move freely in the plane. Graphene and graphene oxide show excellent electrical, mechanical, and thermal properties due to their unique structural and morphological features.^30–36^ They exhibit the Hall effect, tunneling effect, bipolar electric field effect and high thermal conductivity. The electrons are not easily scattered when they are transported in *G*, and the maximum electron mobility at room temperature can reach 2 × 10^5^ cm^2^ (V s)^−1^,^24^ and thus the ideal conductivity of *G* is over 1 × 10^6^ S cm^−1^.^24^ The Young's modulus of *G* can reach up to 1100 GPa,^25^ its transmittance is about 97.9% for visible light,^25^ and its specific surface area can be as high as 2630 m^2^ g^−1^.^26^ As a two-dimensional carbon material, graphene oxide has a single atomic layer, and the size of its sheets is polydisperse. Compared with graphene, there are many oxygen-containing functional groups on the graphene oxide sheet layer, thus the structure of graphene oxide is more complicated, and its properties depend on its structure. Lerf and Klinowski^37,38^ put up the structural model of graphene oxide, called the L–K model. It indicates that the hydroxyl and epoxy groups are randomly distributed on the graphene oxide single layer, while the carboxyl and carbonyl groups are introduced at the edge of the single layer. Graphene oxide has an unoxidized benzene ring region and an oxidized aliphatic six-membered ring region, and the relative size of these two regions depends on the degree of oxidation and random distribution on graphene oxide. However, the L–K structural model is based on certain conditions, ignoring the influence of raw graphene, oxidant and oxidation methods. Erickson *et al.*^39^ studied graphene oxide nano-plates *via* scanning electron microscopy (SEM), and found that graphene oxide not only has an oxidized region with high disorder and unoxidized graphene region, but also has hole defects due to overoxidation and lamellar peeling. Accordingly, in recent years, other models have been proposed, such as the dynamic structure model (DSM)^40,41^ and binary structure model.^42^ Extensive research has been conducted on the preparation and properties of G and GO by many scientists, and it has been found that the obvious difference between graphene and graphene oxide is the addition of oxygen atoms bound with some carbons, as shown in Fig. 2. As a result, graphene is hydrophobic in nature, whereas graphene oxide is hydrophilic and easily dispersed in water. In addition, graphene oxide contains both aromatic (sp^2^) and aliphatic (sp^3^) domains, which lead to an increase in the type of interactions that can occur on its surface. Graphene oxide can be reduced to graphene by a reducing agent. However, the produced graphene is not suitable for electronic applications and mechanical reinforcement of polymers due to the structural defects created during the synthesis of graphene oxide. Nevertheless, this is the preferable route for the large-scale modification of the surface properties of graphene materials by functionalization.

## 3. Preparation of graphene oxide

**Fig. 3:**
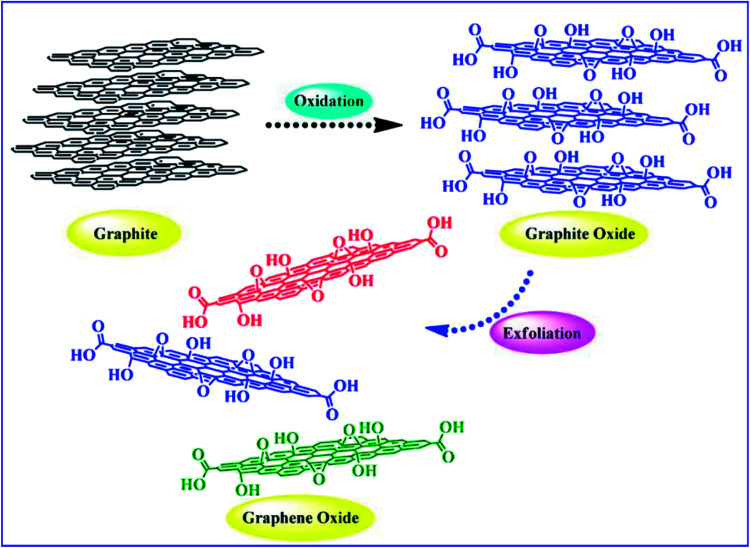
Preparation of graphene oxide.

**Fig. 4:**
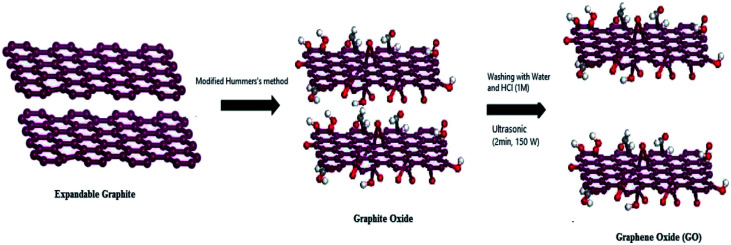
Schematic synthesis of graphene oxide by the modified Hummers' method. This figure has been reproduced from ref. 53 with permission from *Chem. Mater.*, Copyright 1999.

The preparation of graphene oxide is generally carried out *via* two steps of oxidant intercalation oxidation and sheet peeling, as shown in Fig. 3. 150 years ago, Brodie^44^ prepared graphite oxide for the first time, but it was not noticed at that time. With the development of the graphene oxide, the main methods for the synthesis of graphene oxide are presented in Table 1. The schematic synthesis of graphene oxide by the modified Hummers' method is shown in Fig. 4.

**Table 1:** The main methods of synthesize graphene oxide

Scientists	Reagent	Reaction time	Reaction temperature	Characteristics
Brodie^44^	KClO_3_, HNO_3_	3–4 h	60 °C	The first method
Staudenmaier^45^	KClO_3_, HNO_3_, H_2_SO_4_	96 h	RT	
Hummers^46^	HNO_3_	20 h	RT	Only using HNO_3_
David^47^	KMnO_4_, NaNO_3_, H_2_SO_4_	<2 h	35 °C	Reaction time is short
Eigler^48^	KMnO_4_, NaNO_3_, H_2_SO_4_	16 h	10 °C	High quality RGO
Peng^49^	K_2_FeO_4_, HSO_4_	1 h	RT	No heavy metal manganese pollution
Marcano^50^	H_2_SO_4_, H_3_PO_4_, KMnO_4_	12 h	50 °C	Low toxicity
Panwar^51^	H_2_SO_4_, H_3_PO_4_, KMnO_4_, HNO_3_	3 h	50 °C	High yield
Shen^52^	Benzoyl peroxide	10 min	110 °C	No liquid

The different methods have advantages and disadvantages. In the early stages, nitric acid and KClO_3_ were used, but harmful gases such as ClO_2_, NO_2_ and N_2_O_4_ were generated during the reaction, which took a long time.^52,54^ The Hummers' method was used widely; however, this method produces NO_2_, N_2_O_4_ and heavy metal pollution. Also, the products contained Na^+^ and NO_3_^−^ ions, which were difficult to remove. Besides, the oxidizing agent (NaNO_3_/KMnO_4_) had strong oxidizing properties in concentrated sulfuric acid, and there was a high risk of explosion when Mn_2_O_7_ contacted with organic matter at 55 °C.^55^ Subsequently, Marcano^50^ used the less corrosive phosphoric acid to produce graphene oxide *in situ*. This method does not involve the release of intense exothermic or toxic gases, and the prepared graphene oxide has a higher degree of oxidation and structural regularity, but requires a large amount of KMnO_4_ and concentrated sulfuric acid, thus increasing the cost of processing raw materials and the treatment of waste liquids. The preparation of graphene oxide using the K_2_FeO_4_/H_2_SO_4_ oxidation system^49^ at room temperature for 1 h is a safe and efficient method. Using benzoyl peroxide^52^ as an oxidant at 110 °C, graphene oxide can also be prepared by reaction for 10 min. Although this method is highly efficient, benzoyl peroxide itself is extremely unstable and there may be a risk of explosion when the reaction temperature is high. Besides the effect of oxidants on graphene oxide, the graphite raw materials and the reaction methods also affect the properties of graphene oxide, where relatively few hydroxyl and carboxyl groups are obtained on graphene oxide prepared from highly crystalline graphite powder.^56^ During the low-temperature and mid-temperature stages of graphene oxide preparation, a modified Hummers' method with the help of auxiliary ultrasound equipment was used, and the layer spacing of graphene oxide became larger.^57^ However, there were many holes in the graphene oxide due to excessive oxidation. When the reaction temperature was over 50 °C, the graphene oxide became unstable. If this reaction occurred at a relatively low temperature, the hole defects of graphene oxide would diminish significantly.^58^ Currently, the feasibility of the industrial preparation of graphene with graphene oxide as the precursor is high, but the existing graphene oxide preparation methods generally have technical problems such as complicated purification steps and many defects. Therefore, compared with mechanically exfoliated high-quality graphene, the prepared reduced graphene oxide is inferior in structure and properties.

The proper functionalization of graphene and graphene oxide prevents agglomeration during the reduction of graphene and graphene oxide,^59^ and preserves their inherent properties. The functionalization of graphene and graphene oxide results in new functions, and subsequently can possess excellent mechanical properties, electrical properties, thermal properties, *etc.* Currently, the methods for the functionalization of graphene and graphene oxide mainly include covalent bond functionalization, non-covalent bond functionalization, and other atomic doping functionalization.^60^

## 4. Reduction of graphene oxide

### 4.1 Sodium borohydride reducing agent

**Fig. 5:**
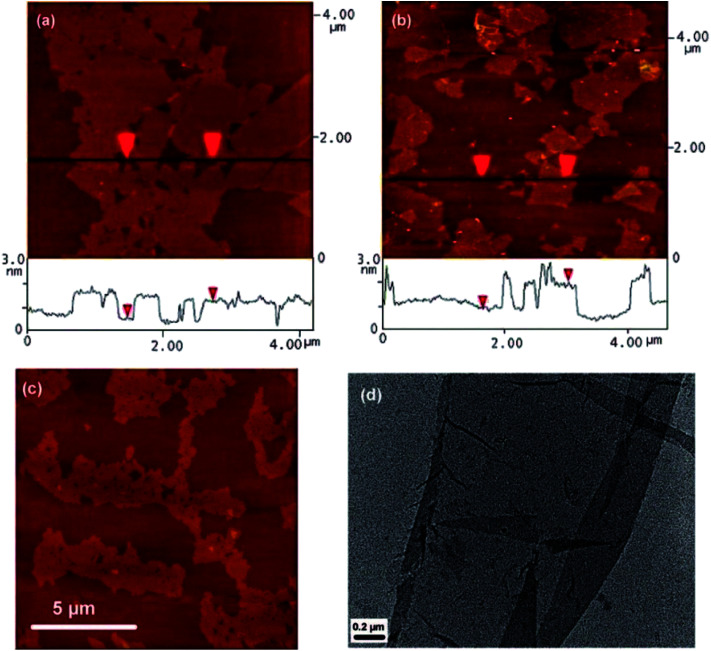
Images of isolated graphene oxide and graphene sheets. (a) AFM image of graphene oxide sheets on freshly cleaved mica, the height difference between two arrows is 1 nm, indicating a single graphene oxide sheet; (b) AFM image of water-soluble graphene on freshly cleaved mica; the height difference between two arrows is 1.2 nm; (c) AFM image of large graphene oxide sheets on mica, small holes in the sheets are caused by over-exposure to sonicaion; (d) TEM image of a partially folded water-soluble graphene sheet. This figure has been reproduced from ref. 61 with permission from *Nano Lett.*, Copyright 2008.

Si *et al.*^61^ prepared graphene from graphene oxide using the sodium borohydride reducing agent and then sulfonation with the aryl diazonium salt of sulfanilic acid. The light sulfonated graphene was easily dispersed in water at a suitable concentration (2 mg mL^−1^) in the pH range of 3–10. By observing the AFM images shown in Fig. 5, they found that the lateral dimension of graphene oxide was several micrometers and its thickness was 1 nm, but there were some differences with the reaction of chemically reduced GO (RGO), where the lateral dimension varied from several hundred nanometers to several micrometers, and the thickness was about 1.2 nm. During the experiment, excessive ultrasonic treatment may cause some small hole-like defects to be introduced in graphene oxide, causing the single graphene sheet to be folded over on one edge with isolated small fragments of graphene on its surface (Fig. 5(d)), which was the reason why the AFM images showed an increase in RGO thickness to 10 μm.

Choi *et al.*^62^ reported a simple method for the fabrication of multifunctional fibers with mechanically strong RGO cores and highly conductive chemical vapor deposition (CVD) graphene shells (rGO@Gr fibers), which showed outstanding electrical conductivity as high as 137 S cm^−1^ and a failure strain value of 21%. These values are believed to be the highest values among polymer-free graphene fibers. We also demonstrated the use of the rGO@Gr fibers in high power density supercapacitors with enhanced mechanical stability and durability, which enables their practical applications in various smart wearable devices in the future. The main method involved the preparation of the CVD graphene film, GO fiber fabrication, fabrication of graphene–GO fiber (a reduction solution (HI : AcOH = 2 : 5 v/v)) and testing the thermal conductivity.

### 4.2 Alcohol reducing agent reduction method

The reduction of GO with an alcohol is a relatively mild reduction method because this treatment method does not cause severe damage to the edge morphology of GO, and highly conductive RGO can be prepared.

Su *et al.*^63^ reduced GO with ethanol vapor at high temperature to obtain highly conductive RGO. Experiments showed that the impedance of GO was 188–418 kΩ μm^−2^, and after ethanol vapor reduction at 900 °C, the impedance was reduced by 43 kΩ μm^−2^. This result was similar to the result from reducing GO at 1100 °C under vacuum (40–100 kΩ μm^−2^). Besides, they also studied the reduction of GO with H_2_ at high temperature. The results indicated that ethanol vapor was more effective at reducing GO at the same temperature.

### 4.3 Phenolic reduction method

In addition to alcohol reducing agents, some phenolic reducing agents are also used to reduce GO. Wang *et al.*^64^ studied the peeling of GO for 20 h into a GNS sheet by hydroquinone upon refluxing GO with hydroquinone. The results showed that the product RGO had a low oxygen content and maintained an ordered crystal structure, but agglomeration occurred within only a few hours in water. It was also found that the thermal stability of the product was worse than that of graphite powder.

### 4.4 Thermal exfoliation and reduction

Thermally reduced graphene oxide (TRG) can be produced *via* the rapid heating of dry GO under inert gas and high temperature. The properties of thermal reduction products are directly related to the heating rate, reduction temperature and time. Too fast heating rate and too long reduction time will greatly expand the volume of the product, and the specific surface area is much lower than the theoretical value of graphene. Hyunwoo Kim^65^ reviewed that in top-down processes, graphene or modified graphene sheets are produced by separation/exfoliation of graphite or graphite derivatives. In general, these methods are suitable for large-scale production for polymer composite applications. For the process of TRG, heating GO in an inert environment for 30 s at 1000 °C causes the reduction and flaking of GO, thereby producing TRG sheets. Delamination occurs when the pressure generated by the gas (CO_2_) due to the decomposition of the epoxy and hydroxyl groups of GO exceeds the van der Waals force holding the graphene oxide sheets together.

Fadil^66^ also reported a polymer/GO material, where its preparation involved aqueous mini-emulsion copolymerization of St and nBA in the presence of nano-dimensional GO (small and large sheets) and the conventional surfactant SDS. The obtained latex comprising polymer particles armoured with GO sheets were used directly for film formation at ambient temperature.

### 4.5 Other methods

**Fig. 6:**
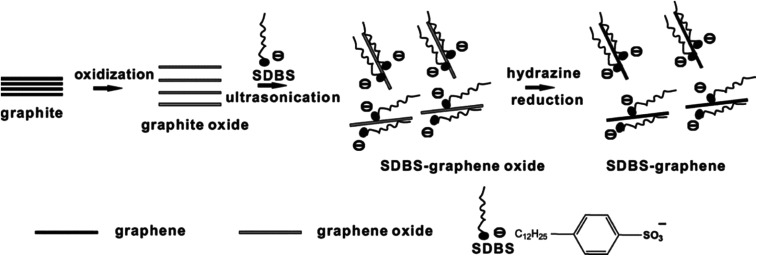
Schematic of solution-processible SDBS–graphene synthesis by *in situ* reduction with SDBS as the stabilizing agent. This figure has been reproduced from ref. 53 with permission from *Nano Lett.*, Copyright 2007.

There as other reduction methods such as sulfur-containing compound reducing agent reduction method, SO_2_ reduction, ultraviolet radiation reduction method, and electrochemical reduction method. Some researchers prepared RGO using sulfur-containing compound reducing agents, and found that sulfur-containing compounds have good reducing properties. The oxygen content in the reduced product is relatively low and stably dispersed in solution. When SO_2_ gas was used as a reducing agent for reducing GO, the oxygen content of the product RGO obtained by this method was similar to that using hydrazine as the reduction agent, and the sulfur-containing compound could promote the dispersion of the product in aqueous solution.^67^ It was also found that the product RGO was a single layer having a thickness of only 0.87 nm, but the surface had significant wrinkles. When the number of layers was below 10 layers, a large area of wrinkles appears in GO,^68^ as shown in Fig. 6.

**Fig. 7:**
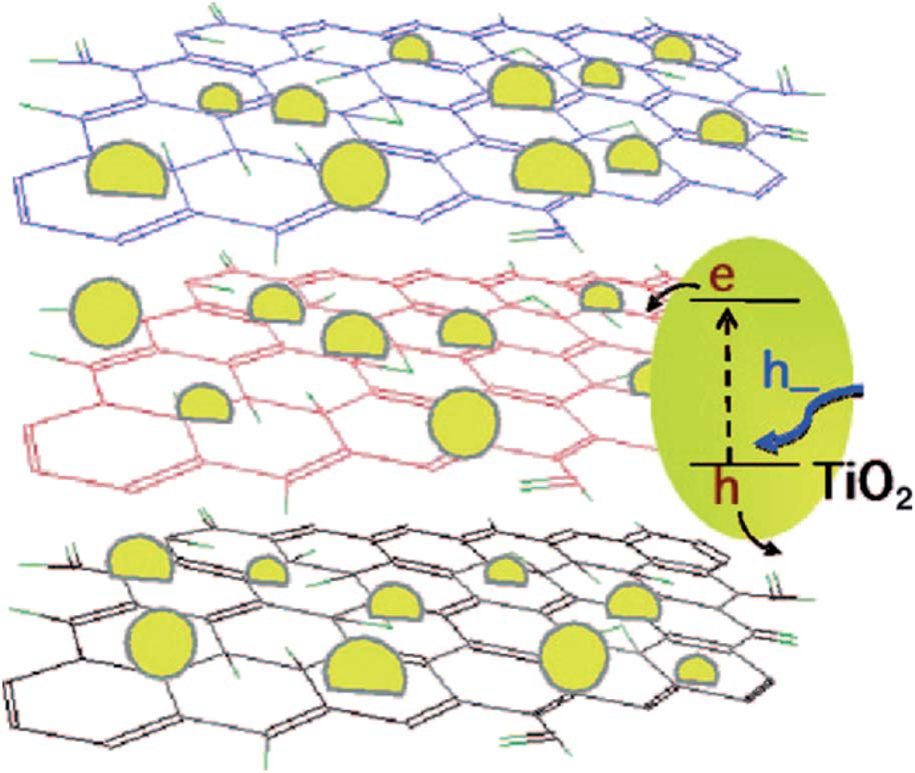
TiO_2_–graphene composite and its response under UV-excitation. This figure has been reproduced from ref. 69 with permission from *ACS Nano*, Copyright 2008.

Ultraviolet radiation reduction and electrochemical methods are also environmentally friendly methods for the preparation of GNS, and they have a great potential in the large-scale preparation of GNS. Williams *et al.*^69^ conducted an in-depth study on the ultraviolet (UV) irradiation reduction method, and a schematic of the TiO_2_–graphene composite and its response under UV-excitation is shown in Fig. 7.

They obtained stable RGO by reducing the GO suspension by UV irradiation. Compared with the use of a photocatalyst or chemical reducing agent, this method is not only simple and easy, but also inexpensive, and it provides a new possible way for the mass preparation of GNS.

## 5. Functionalized graphene and graphene oxide

The proper functionalization of graphene and graphene oxide prevents their agglomeration during the reduction process,^59^ and preserves their inherent properties. The functional modification of graphene and graphene oxide not only maintains their excellent characteristics, but also introduces new functional groups to give them new characteristics. Also, different functional groups give different characteristics. Presently, the methods for the functionalization of graphene and graphene oxide mainly include covalently functionalization, non-covalent functionalization, and elemental doping.^70^

### 5.1 Covalent functionalization

Covalent bond functionalization of graphene involves combining graphene with newly introduced groups in the form of covalent bonds to improve and enhance its performance. The oxygen-containing groups on the surface of graphene oxide makes covalent bond functionalization easier than that on graphene. The surface of graphene oxide contains a large amount of hydroxyl groups, carboxyl groups, and epoxy groups. These groups can be used for common chemical reactions such as isocyanation, carboxylic acylation, epoxy ring opening, diazotization, and addition.^71^ The covalent bond functional modification of graphene and graphene oxide is illustrated below according to the functional group. Covalent bond modification increases the processability and brings new functions. There are multiple strategies to covalently functionalize GO. For example, Man *et al.*^72^ using polystyrene particles “armoured” with nanosized graphene oxide (GO) sheets, which were prepared by aqueous mini-emulsion polymerization of styrene, exploiting the amphiphilic properties of GO in the absence of conventional surfactants. The nanoscale GO sheets were prepared from graphite nanofibers with a diameter of approximately 100 nm based on a novel procedure, thus effectively ensuring the absence of larger sheets.

#### 5.1.1 Carbon skeleton functionalization

The functional modification of the carbon skeleton is mainly carried out using the C=C bond in the aromatic ring of graphene or graphene oxide. The graphene oxide diazotization reaction and Diels–Alder reaction have been reported.^73–75^

**Fig. 8:**
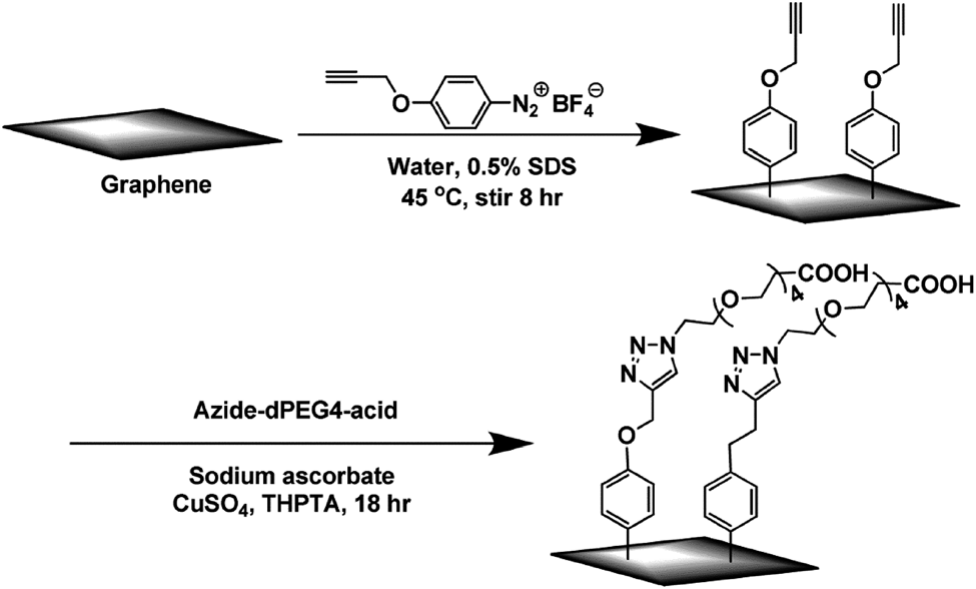
Diazonium reaction and subsequent click chemistry functionalization of graphene sheets. This figure has been reproduced from ref. 77 with permission from *Chem. Mater.*, Copyright 2011.

Zhong *et al.*^76^ used solution-phase graphene as a raw material dispersed in 2% sodium cholate (as surfactants) aqueous solution, and stirred it with 4-propargyloxydiazobenzenetetrafluoroborate at 45 °C for about 8 h, to obtain 4-propargyloxyphenyl graphene (GC≡CH). Then, a click chemistry reaction with the azido polyethylene glycol carboxylic acid (as shown in Fig. 8) was carried out to realize an addition reaction to the graphene carbon skeleton, thereby further functionalizing the graphene. This method is flexible and convenient, and can be used to prepare graphene composite materials and biosensors by changing the functionalized modified groups connecting graphene.

This, it can be concluded that the basic process is a diazonium salt or a diazo compound formed by diazotization of an aromatic amine-containing substance having a reactive functional group, and an electron is removed to form a free radical after deaeration.^77^ Then, a double bond addition reaction with C=C is carried out to form a new C–C single bond, which is linked to the derivative of benzene having a reactive functional group by a sigma bond, distributed on the surface, and then further, graphene with is reactive functional groups undergo functional modification with graphene oxide.

#### 5.1.2 Hydroxy functionalization

**Fig. 9:**
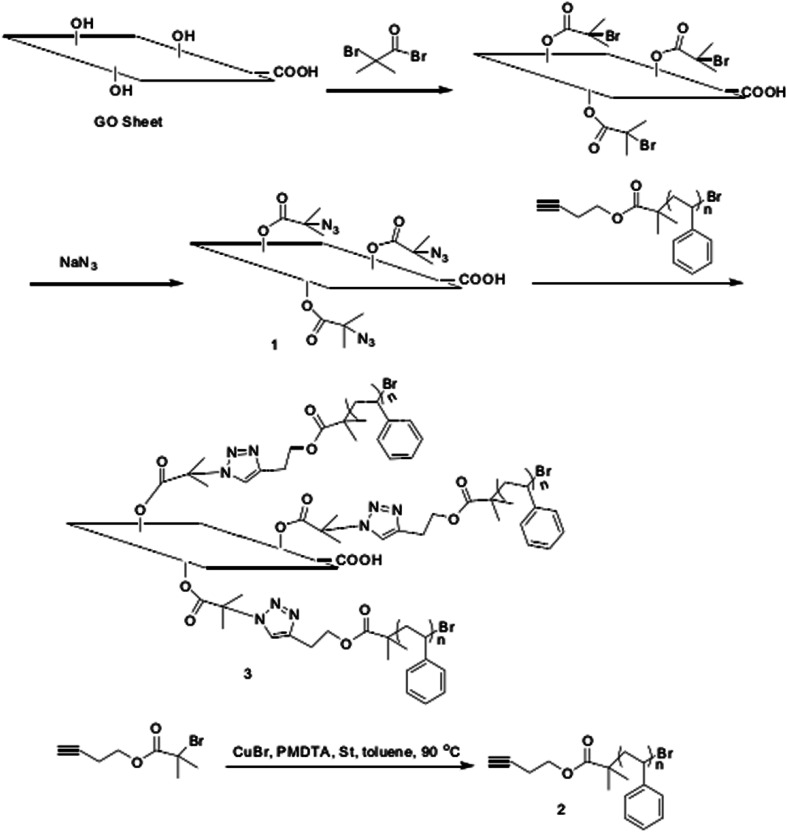
Synthetic route of polystyrene graft graphite oxide (GO/PS). This figure has been reproduced from ref. 80 with permission from *Polymer*, Copyright 2011.

Graphene oxide contains a large amount of reactive hydroxyl groups on its sheet layer, where hydroxyl-based functional modification generally involves hydroxy-reaction of amide or isocyanate and graphene oxide to produce esters, and then further functional modification using different groups.^78^ Yang *et al.*^79^ prepared azidolated graphene oxide *via* esterification and substitution of the hydroxyl groups on the surface of graphene oxide. Firstly, graphene oxide and 2-bromoisobutyryl bromide were stirred at room temperature for 48 h. After esterification, the product was dispersed in dimethylformamide, and NaN_3_ was added at room temperature for 24 h to obtain azido-modified graphene oxide (GO–N_3_). Finally, alkyne-functionalized polystyrene (HC≡C–PS) was used to graft polystyrene onto the surface of the oxide by esterification to obtain graphene-based polystyrene. The modified graphene oxide had good solubility in polar solvents such as tetrahydrofuran, dimethylformamide and chloroform, and the distance between GO layers could be controlled by the length of PS. This method can be extended to the functionalization of other graphite polymer composites. The preparation route and reaction conditions for the target hydroxy-based functionalization of graphene oxide is shown in Fig. 9.

**Fig. 10:**
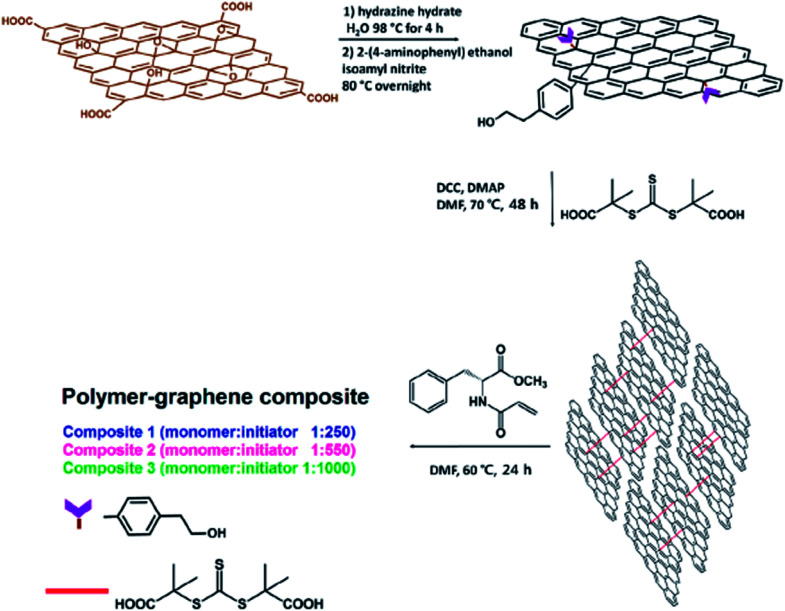
Preparation of polymer–graphene composites. This figure has been reproduced from ref. 81 with permission from *J. Colloid Interface Sci.*, Copyright 2017.

Namvari *et al.*^80^ demonstrated novel reversible addition–fragmentation chain transfer agent (RAFT-CTA)-modified reduced graphene oxide nanosheets (CTA-rGONSs) by crosslinking rGONSs with a RAFT-CTA *via* esterification reaction. These nano CTA-rGONSs were used to polymerize a hydrophobic amino acid-based methacrylamide (*N*-acryloyl-l-phenylalanine methyl ester) monomer with different monomer/initiator ratios. The synthetic procedure is shown in detail in Fig. 10.

#### 5.1.3 Carboxyl functionalization

**Fig. 11:**
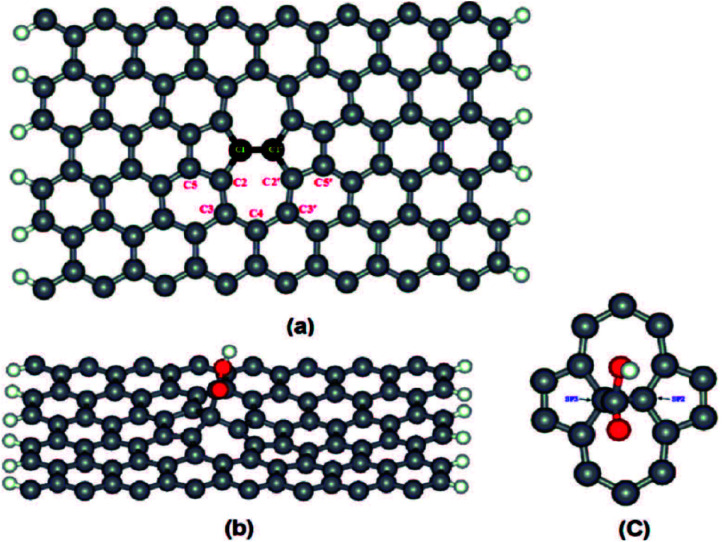
Preparation route and reaction conditions for the target hydroxy-based functionalization graphene oxide. This figure has been reproduced from ref. 88 with permission from *J. Colloid Interface Sci.*, Copyright 2017.

There are a large number of carboxyl groups at the edge of graphene oxide, and the carboxyl group is a highly reactive group, and thus has been greatly studied for the functionalization of graphene oxide.^81,82^ The carboxyl functionalization step is generally the activation of the reaction, and then the group containing an amino group and a hydroxyl group is dehydrated to form an ester or amide bond. The reagents commonly used for carboxyl activation include thionyl chloride (SOCl_2_),^83^ 2-(7-aza-1*H*-benzotriazole-1-yl)-1,1,3,3-tetramethyluronium hexafluorophosphate,^84^*N*,*N*-dicyclohexylcarbodiimide (DCC),^85^ and 1-ethyl-3-(3-dimethylaminopropyl)-carbodiimide (EDC).^86^ Ouyang *et al.*^87^ demonstrated the chemical functionalization of semiconducting graphene nanoribbons (GNRs) with Stone–Wales (SW) defects by carboxyl (COOH) groups. It was found that the geometrical structures and electronic properties of GNRs changed significantly, and the electrical conductivity of the system could be considerably enhanced by mono-adsorption and double adsorption of COOH, which sensitively depends on the axial concentration of SW defect COOH pairs (SWDCPs). With an increase of the axial concentration of SWDCPs, the system transformed from semiconducting behavior to p-type metallic behavior. This makes GNRs a possible candidate for application in chemical sensors and nanoelectronic devices. The preparation route and reaction conditions for the target hydroxy-based functionalization of graphene oxide are shown in Fig. 11.

**Fig. 12:**
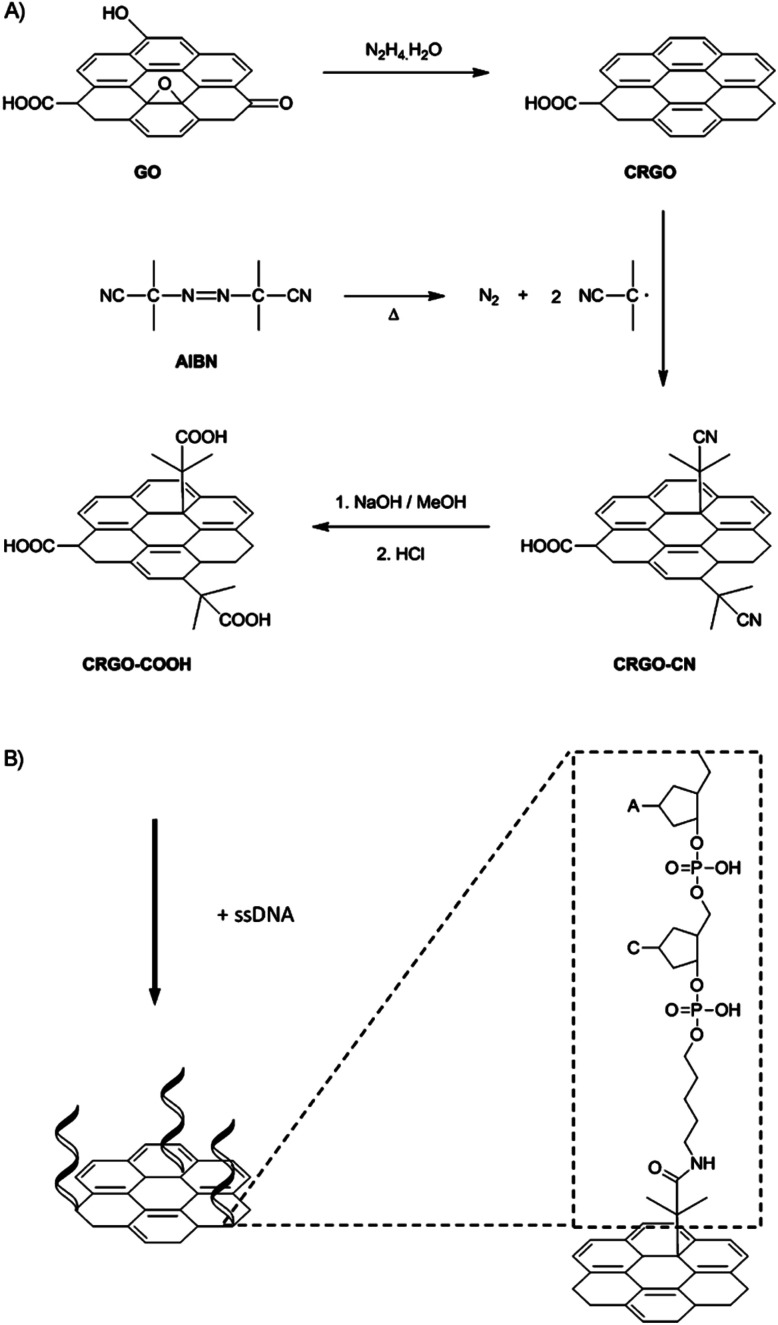
Schematic of the functionalization method. This figure has been reproduced from ref. 89 with permission from *Chem.–Eur. J.*, Copyright 2014.

Bonanni *et al.*^88^ removed the oxygen-containing groups from GO through a reductive treatment, and consequently reintroduced carboxyl groups onto the graphene surface. Carboxyl groups were inserted based on a free-radical-addition reaction, which occurred not only on the carbon atoms located at the edge plane, but also at the much more abundant basal plane of the graphene sheets. Specifically, chemically reduced graphene oxide (CRGO) was first functionalized with isobutyronitrile groups, which were generated from the thermal decomposition of azobisisobutyronitrile (AIBN) to give CRGO–CN. Subsequent reflux of CRGO–CN in a mixture of methanol and sodium hydroxide aqueous solution resulted in a hydrolysis reaction to provide CRGO enriched with carboxyl groups (CRGO–COOH), as shown in Fig. 12.

### 5.2 Non-covalent functionalization

The non-covalent bond functionalization of graphene or graphene oxide results in the formation of a composite material having a specific function by interaction between hydrogen bonds and electrostatic forces between graphene and functional molecules, the greatest advantage of which is maintaining the bulk structure and excellent properties of graphene or graphene oxide, and also improving the dispersibility and stability of graphene or graphene oxide. The methods for the functional modification of surface non-covalent bonds mainly include π–π bond interaction, hydrogen bonding, ionic bonding, and electrostatic interaction modification. The non-covalent bond functionalization process is simple with mild conditions, while maintaining the structure and properties of graphene. However, the disadvantage of this method is that other components (such as surfactants) are introduced.

#### 5.2.1 π–π bond interaction

Song *et al.*^89^ inspired by interfacial interactions of protein matrix and the crystal platelets in nacre, produced a super tough artificial nacre through the synergistic interface interactions of p–p interaction and hydrogen bonding between graphene oxide (GO) nanosheets and sulfonated styrene–ethylene/butylene–styrene copolymer synthesized with multifunctional benzene. The resultant GO-based artificial nacre showed super-high toughness of 15.3 ± 2.5 MJ m^−3^, superior to that of natural nacre and other GO-based nanocomposites. The ultra-tough property of the novel nacre was attributed to the synergistic effect of the p–p stacking interactions and hydrogen bonding. Thus, this bioinspired synergistic toughening strategy opens a new avenue for constructing high performance GO-based nanocomposites in the near future.

**Fig. 13:**
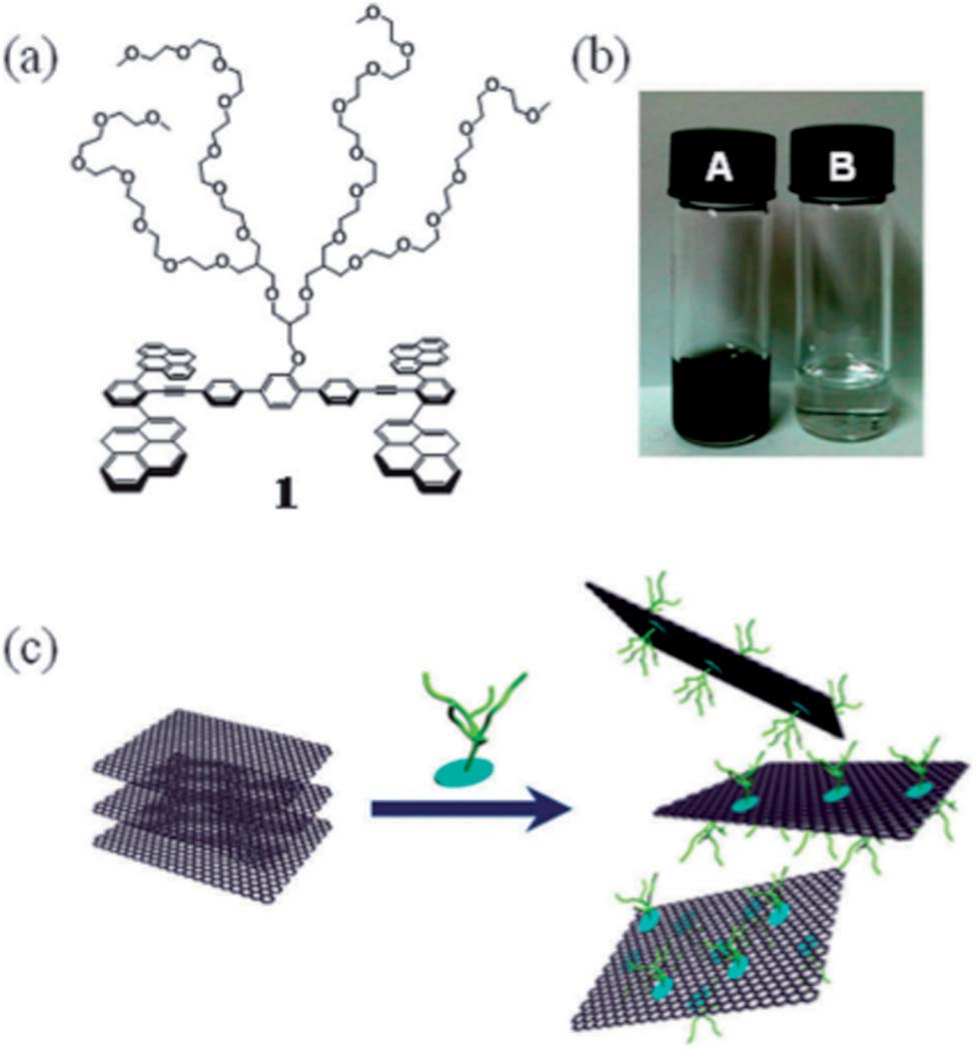
Exfoliation process of graphite and stabilization of graphene though the π–π interaction with tetrapyrene derivative. This figure has been reproduced from ref. 91 with permission from *R. Soc. Chem.*, Copyright 2016.

Lee *et al.*^90^ used a tetradecene derivative with a dendritic polyether branch as a modifier and the synergistic effect of an aromatic cyclic fluorene skeleton interacting with graphite and a polyether chain to induce high hydrophilicity, stripping graphite and stabilizing the graphene layer, as shown in Fig. 13. The same indole derivative did not improve the dispersibility of single-walled carbon nanotubes, indicating that the planar structure of carbon nanomaterials is a key factor in the formation of effective π-stacking. Due to the π–π bond, the absorption spectrum of tetraterpene derivatives appeared red shifted and their fluorescence was also quenched.

#### 5.2.2 Hydrogen bond interaction

He *et al.*^91^ researched the fabrication of reduced graphene oxide aerogel membranes (rGOAMs) *via* the reduction-induced self-assembly of rGO through hydrogen bond mediation. Using polyethylene glycol (PEG) as the mediator, the PEG–rGO hydrogen bonding interactions partly replaced the interlayer p–p and hydrophobic interactions during reduction, decreasing the rGO laminate size in 2D stacking and alleviating the structural shrinkage of the rGOA networks. The tight correlation between membrane pore size and porosity was broken, leading to rGOAMs with tunable pore sizes (0.62 to 0.33 mm) and high porosity (95%). The resultant rGOAMs could effectively reject oil-in-water emulsions with different sizes and exhibited ultrahigh water flux (up to 4890 L m^−2^ h^−1^) under 0.10 bar as well as a persistent anti-oil-fouling performance for up to 6 cycles.

**Fig. 14:**
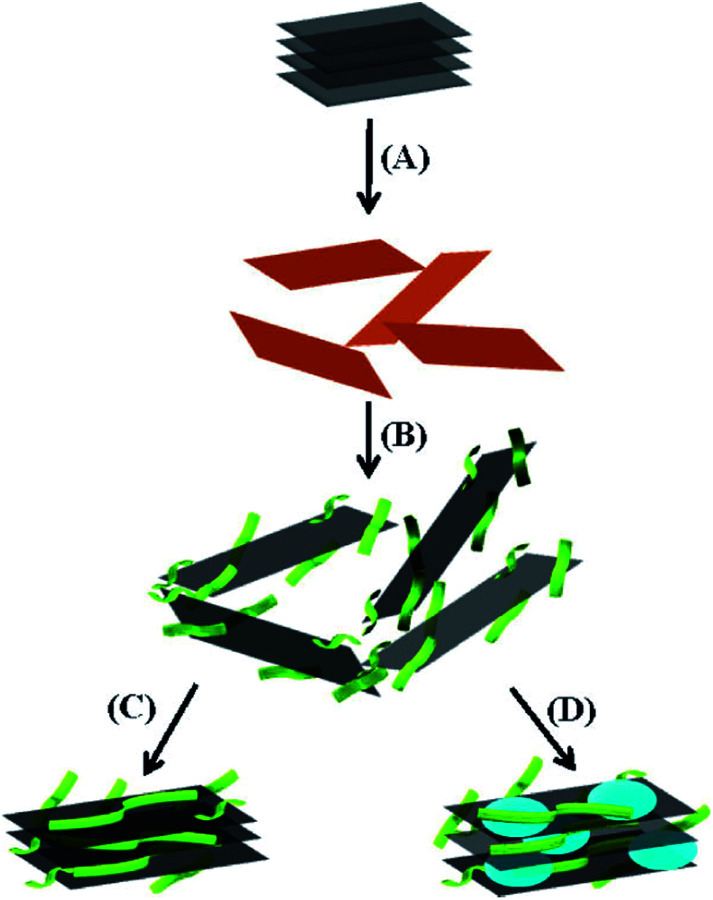
Schematic illustration of aqueous dispersions of (A) GO, (B) GO–PDI and (C) GO–PyS through π–π interaction and (D) co-assembly of negatively charged ssDNA-G sheets and positively charged cytochrome *c* produces co-intercalated multifunctional layered nanocomposites. This figure has been reproduced from ref. 93 with permission from *Adv. Mater.*, Copyright 2009.

Patil *et al.*^92^ realized the surface functionalization of graphene by hydrogen bonding between graphene and DNA, which improved the hydrophilicity of graphene and stabilized it in water. On the other hand, the loading of organic molecules occurred on the surface of graphene. The functional modification of the graphene surface by hydrogen bonding does not introduce impurities, which is safe and reliable, and has important potential application prospects in biomedical field. A schematic illustration of aqueous dispersions of (a) GO, (b) GO–PDI and (c) GO–PyS through π–π interaction is shown in Fig. 14.

**Fig. 15:**
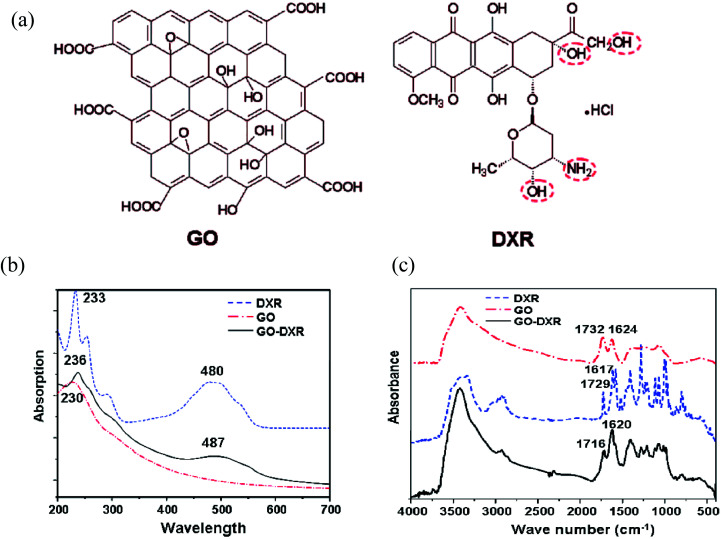
(a) Structure of graphene oxide (GO) and DXR, UV visible spectra (b) and FTIR spectra (c) of DXR, GO, and GO–DXR. This figure has been reproduced from ref. 94 with permission from *Nano Lett.*, Copyright 2011.

A novel graphene oxide–doxorubicin hydrochloride nanohybrid (GO–DXR) was prepared *via* a simple noncovalent method, and the loading and release behaviors of DXR on GO were investigated by Patil *et al.*^93^ The efficient loading of DXR on GO was as high as 2.35 mg mg^−1^ at the initial DXR concentration of 0.47 mg mL^−1^. The loading and release of DXR on GO showed strong pH dependence, which may be due to the hydrogen-bonding interaction between GO and DXR. The fluorescent spectrum and electrochemical results indicate that strong π–π stacking interaction exists between them, as shown in Fig. 15.

#### 5.2.3 Ion interaction

**Fig. 16:**
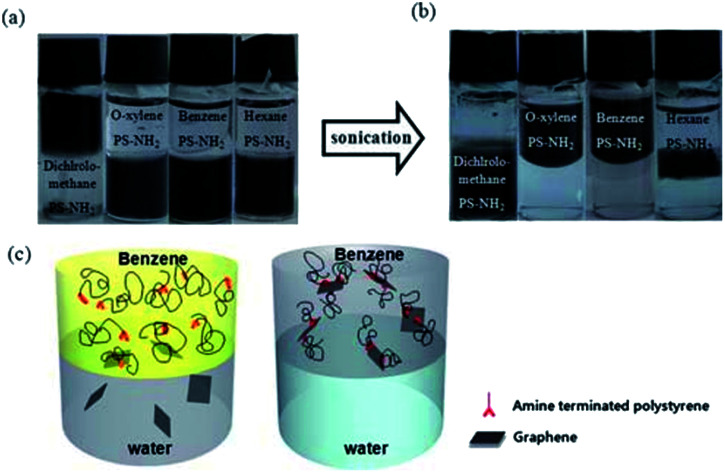
Transformation of the hydrophilic rGO into an organophilic rGO/polymer composite using an amine terminated polystyrene. This figure has been reproduced from ref. 95 with permission from *J. Mater. Chem.*, Copyright 2010.

Choi *et al.*^94^ reported that a stable dispersion of reduced graphene was achieved in various organic solvents *via* noncovalent ionic interaction functionalization with amine-terminated polymers. An aqueous dispersion of reduced graphene was prepared *via* the chemical reduction of graphene oxide in aqueous media and vacuum filtered to generate reduced graphene sheets. Thorough FTIR and Raman spectroscopy investigation verified that the protonated amine terminal group of polystyrene underwent noncovalent functionalization with the carboxylate groups on the graphene surface, providing high dispersibility in various organic media, as shown in Fig. 16.

Ge *et al.*^95^ improved the mechanical and barrier properties of a starch film with reduced graphene oxide (RGO) modified by sodium dodecyl benzene sulfonate (SDBS). The hydrophilia of the modified RGO (r-RGO) was improved, which resulted in its good dispersion in the oxidized starch (OS) matrix. The tensile strength of the r-RGO-4/OS film increased to 58.5 MPa, which was more than three times that of the OS film (17.2 MPa). Besides, both the water vapor and oxygen barrier properties of the r-RGO/OS film improved greatly compared with that of the OS and GO/OS films. Moreover, the r-RGO/OS film could protect against UV light effectively due to its lightproof performance. In conclusion, the r-RGO/OS composite film has great potential applications in the packaging industry.

#### 5.2.4 Electrostatic interaction

**Fig. 17:**
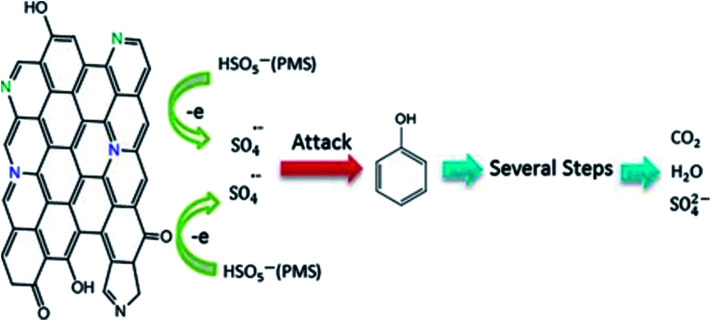
Synthesis of well-dispersed graphene by electrostatic repulsion. This figure has been reproduced from ref. 96 with permission from Nature Publishing Group, Copyright 2008.

Electrostatic repulsion between the same type of charge is another strategy to improve the dispersion of graphene. Bhunia *et al.*^60^ used hydrazine as a reducing agent to control the reduction, while removing the functional groups such as hydroxyl groups and epoxy bonds of graphene oxide and retaining the carboxyl anion, which is well dispersed by charge repulsion. The chemical conversion of graphene can be conducted in water. Graphene oxide is soluble in water because its surface negative charge repels each other and forms a stable colloidal solution, as shown in Fig. 17.

Ge X. and Li H.^96^ prepared novel environmentally friendly Gemini surfactants, *i.e.* each with two hydrophilic and two hydrophobic groups. The reaction was conducted in hydrogen peroxide for the epoxidation of the carbon–carbon double bond. Methylene was used as the spacer group, and the nonionic hydrophilic head group was then introduced by ring-opening reaction. The targeted surfactants were synthesized *via* the reaction of chlorine sulfonic acid and hydroxy esterification. The results were compared with that for mixtures of the standard surfactants sodium decylsulfate and octaoxyethyleneglycol mono *n*-decyl ether under equivalent conditions. The surfactants were shown to exhibit improved performance over the mixed system both in terms of micellization and surface tension lowering.

### 5.3 Element doping

Element doping modification usually adopts annealing heat treatment, ion bombardment, arc discharge and other means to incorporate different elements into graphene, thereby resulting in the substitution of defects and vacancy defects in graphene, and maintaining the intrinsic two-dimensional structure of graphene. Simultaneously, its surface properties change to give new performances.^97–100^ Element doping adjusts the energy band structure of graphene, but the doping process is difficult to control quantitatively.

**Fig. 18:**
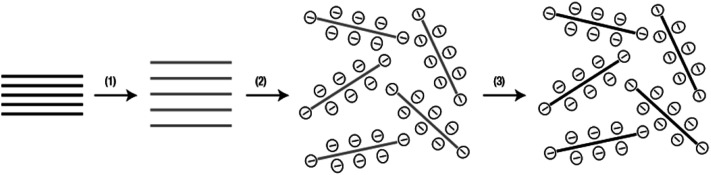
Detailed growth process of N-doped graphene by thermal annealing treatment. This figure has been reproduced from ref. 101 with permission from *Catalysis*, Copyright 2015.

Duan *et al.*^100^ used thermal annealing to treat graphene and ammonium nitrate to prepare 6.54 at% nitrogen-doped graphene, which catalyzed the oxidative degradation of phenol by 5.4 times that of undoped graphene. The synergistic effect of B-, P- or N-doped graphene was studied. The detailed growth process of N-doped graphene by thermal annealing treatment is shown in Fig. 18.

**Fig. 19:**
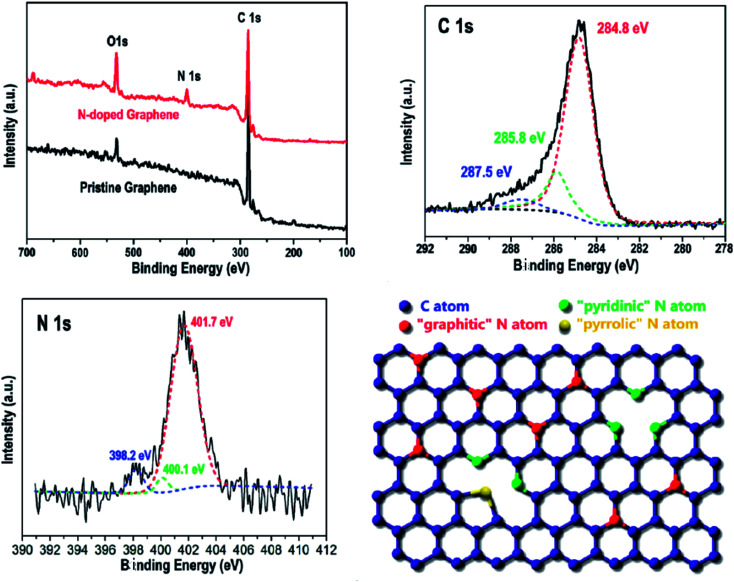
Synthesis of N-doped graphene by electrostatic repulsion. This figure has been reproduced from ref. 102 with permission from *Nano Lett.*, Copyright 2009.

Wei *et al.*^101^ reported a CVD technique to produce N-doped graphene, which was the first experimental example of substitutionally doped graphene, which was hard to produce using other methods. The CVD method is a nondestructive route to produce graphene and realizes substitutional doping since the doping is accompanied by the recombination of the carbon atoms in graphene during the CVD process. By using SEM, TEM, Raman, XPS and EDS, they demonstrated the existence of N-doped graphene. The CVD method can not only be used to produce N-doped graphene, but also has potential to produce the graphene doped with other elements. Moreover, they measured the electrical properties of the N-doped graphene. This research provides a new type of graphene experimentally, which is required for the further application of graphene. The synthesis of N-doped graphene by electrostatic repulsion is shown in Fig. 19.

## 6. Summary of the performance and application of functionalized graphene

The functional modification of graphene is of great significance for the application of graphene composites. According to previously mentioned literature, the surface functional modification of graphene and graphene oxide is used to obtain related products and the interactions and reactions of the modification types have been studied, briefly explaining the functional characteristics and application fields of graphene surface-functionalized composites, as summarized in Table 2. Obviously, this table will be supplemented and improved as the research on graphene continues.

**Table 2:** Properties and applications of functionalized graphene and graphene oxide

Modification type	Modified group	Modification agent	Interaction type	Property	Application	Ref.
Covalent functionalization	–C=C–	4-Propargyloxydia-zobenzenetetrafluoroborate	Diazotization	Water soluble	Biosensors	77
	–OH	2-Bromoisobutyryl bromide, NaN_3_, HC≡C–PS	Esterification	Good solubility	Polymer composites	79
	–COOH	SOCl_2_	Esterification	Conductive	Conducted membrane	84
	–OH	N_2_H_4_, DNA	Addition esterification	Good solubility	Biosensors	89
Non-covalent functionalization	Carbon six-membered	Sulfonated styrene–ethylene/butylene–styrene copolymer	Copolymerization	Conductive	Nanocomposites	90
	Carbon six-membered ring	Tetrapyrene derivative	π–π	Stable and dispersed, conductive	Sensors	91
	–OH	DNA	Hydrogen bond interaction	Stable and dispersed, good solubility	Biomedicine	93
	–OH	DXR	Hydrogen bond interaction	Stable and dispersed, good solubility	Drug carriers	94
	–COOH	Amine-terminated polymers	Ion interaction	Stable and dispersed, good solubility	—	95
	–COOH	SDBS	Ion interaction	Stably dispersed, conductive	Packaging	96
	–COO–	Hydrazine	Electrostatic interaction	Stably dispersed	—	71
Element doping	–C–	B, P, and N	—	Band structure change	Electronic devices	101

## 7. Future prospects

Graphene has many unique and prominent physical and chemical properties (large specific surface area, high transparency and excellent mechanical/thermal/electrical/electrochemistry properties) due to its special structure, and thus has wide application potential in many fields, such as heterojunction solar cells,^102^ medicine,^103^ energy,^104^ water splitting, biosensing, bioimaging, environmental studies, catalysis, photocatalysis, and biomedical technologies.^105,106^ However, a coin has two sides. Because of the unique structure of graphene, it is difficult to disperse in water and organic solvents, which is known as the agglomeration phenomenon of graphene. This problem limits the application of graphene. Therefore, it is important to functionalize graphene and graphene oxide to expand their various applications.

Currently, the study of these functional methods is still in the experimental stage, and researchers are mainly focused on the development of new product varieties and their characterization and application. However, there are several problems as follows. (1) Functionalized target groups difficult to control, (2) the synthetic procedures are complicated, and (3) separation and purification are very difficult.

Thus, considering the above-mentioned superior performance, we should pay attention to the relationship between the structure and properties of graphene and graphene oxide in future work. For example, according to the quantitative structure and property relationship principle, functional groups are introduced into molecules based on functionalized target group design. Functionalized target group design is carried out by molecular simulation technology, and the structure property model is set up to guide the synthesis of new products. However, the synthetic process of different types of graphene and graphene oxide is complex, which involves the introduction of many other special groups, thus it is difficult to control the reaction and obtain the target products. Also, some reaction conditions are extremely harsh. Nevertheless, considering the optimization of reaction procedures and environmentally friendly synthetic routes, graphene and graphene oxide will achieve industrialization. Of course, other new functional modification methods should be developed, such as non-alkali carbonylation and carbon–carbon multi-bond addition reactions, which are rarely reported in functional applications.

## 8. Concluding remarks

Graphene-based composites are involved in many fields with the development of science, such as graphene-based drug carriers to improve the stability, toxicity, metabolic kinetics of drugs, and many aspects are still unclear, pending further research and exploration by scientists. Accordingly, graphene and graphene oxide will be researched completely and use widely in the future.

## Conflicts of interest

There are no conflicts to declare.

## Supplementary Material
